# The complete mitochondrial genome sequence of Himalayan toad *Duttaphrynus himalayanus* (Anura: Bufonidae)

**DOI:** 10.1080/23802359.2020.1715287

**Published:** 2020-01-20

**Authors:** Jiahong Liao, Min Tang, Liqing Peng, Lichun Jiang, Zhangqiang You, Wei Chen

**Affiliations:** Ecological Security and Protection Key Laboratory of Sichuan Province, Mianyang Normal University, Mianyang, China

**Keywords:** Bufonidae, *Duttaphrynus himalayanus*, mitochondrial genome

## Abstract

In this study, the complete mitochondrial genome of *Duttaphrynus himalayanus* was sequenced adopting Illumina high-throughput sequencing method. The complete mitogenome of the species was 17,172 bp in length, including 13 protein-coding genes, 22 transfer RNA (tRNA) genes, two ribosomal RNA genes, and a non-coding control region (CR). The overall base composition of mitogenome was 29.7% A, 29.6% T, 26.0% C, and 14.7% G. Most mitochondrial genes are encoded on the heavy strand, only ND6 and eight tRNA genes on the light strand. The complete mitogenome of *D. himalayanus* can provide an important data for future studies on phylogenetic relationship and population genetics of this species.

Himalayan toad *Duttaphrynus himalayanus* belongs to the family Bufonidae, which is mainly distributed in Pakistan, India, Nepal, Bhutan, Sikkim, and Tibet of China (Fei et al. 2010; Li et al. 2010). It mainly inhabits in mountains with an altitude of 1680–2800 m. In this study, we collected the toad in Chayu county of Tibet (28°29′15.60″N, 97°00′15.12″E; 1805 m in altitude) China and sequenced the complete mitochondrial genome of this species. The specimen was kept in the museum of life science and technology of Mianyang Normal University (specimen code MNU20190418).

The complete mitochondrial genome of *D. himalayanus* is 17,172 bp with the GenBank accession No. MN411629.1 included 13 typical vertebrate protein-coding genes, 22 transfer RNA (tRNA) genes, two ribosomal RNA (rRNA) genes, and one control region, which is similar to the other vertebrate mitochondrial genome (Jiang et al. 2016, 2017). The overall base composition is A: 29.7%, T: 29.6%, C: 26.0%, and G: 14.7%, and the AT content is 58.6%. Except for ND6 and eight tRNA genes which were encoded on the light strand, most mitochondrial genes are encoded on the heavy strand.

For 13 mitochondrial protein-coding genes, nine genes (COXII, ATP8, COXIII, ND3, ND4, ND4L, ND5, ND6, and Cytb) share the start codon ATG, two genes (ND1and ND2) have the start codon ATT, COX1 and ATP6 have start codon GTG and ATA, respectively. Six protein-coding genes (ND1, ND2, COXII, COXIII, ND3, and ND4) are inferred to terminate with an incomplete stop codon T––, four genes (COXI, ATP8, ATP6, and ND4L) show the common termination codon TAA, and three genes (ND5, ND6, and CYTB) use AGG as a stop codon. The A + T content of 13 protein-coding genes ranges from 53.94% to 62.42%, which is higher than that of G + C. In addition, we found that 39 nucleotides from 12 locations overlapped between neighbouring genes with the length of overlapped sequence of 1–17 bp, whereas there are totally 84 bp intergenic nucleotides in seven locations with the length of intergenic spacer of 1–35 bp. Nineteen pairs of genes are directly adjacent without intergenic or overlapping nucleotides. 12S rRNA (933 bp) and 16S rRNA (1602 bp) are located between tRNA-Phe and tRNA-Leu and separated by tRNA-Val gene. The lengths of 22 tRNA genes range from 65 to 73 bp. The putative CR (1760 bp in length) were bound by Cytb and tRNA-Leu. A NJ phylogenetic tree based on the complete mitochondrial genome’s analyses of toads of Bufonidae suggested that *D. himalayanus* has close relationship with *Bufo melanostictus* (Figure 1).

**Figure 1.**
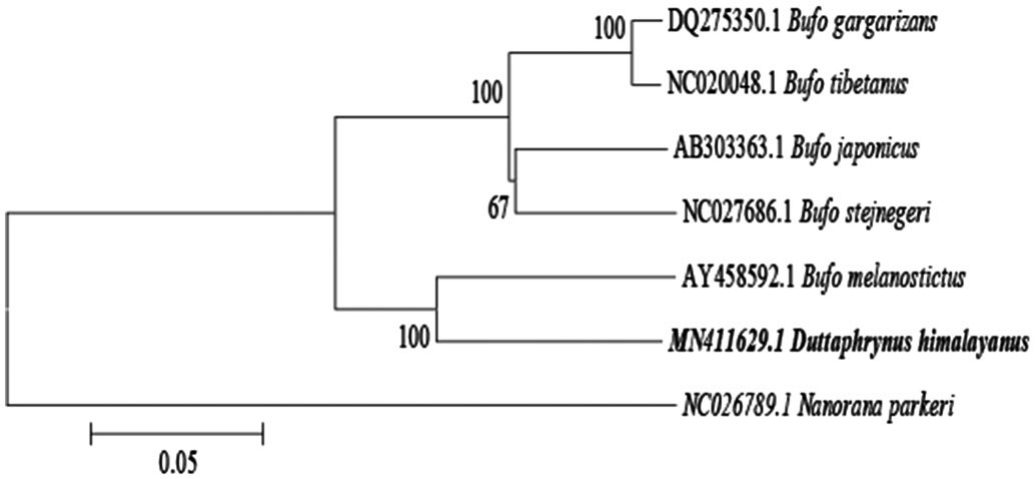
Neighbour-Joining phylogenetic tree of Bufonidae representatives produced based on complete mitochondrial genomes. Genbank accession numbers and bootstrap values of nodes are shown on the tree.

In this study, the whole nucleotide sequence of the *D. himalayanus* was explored and these data will contribute to the phylogenetic relationships and population genetics analysis of Bufonidae in the future.
